# The complete chloroplast genome sequence of *Buxus megistophylla* Levl. (Buxaceae Dumort.)

**DOI:** 10.1080/23802359.2021.1966332

**Published:** 2021-08-22

**Authors:** Xiaoyan Yao, Xiaoxi Meng, Fei Meng, Jing Zhang, Jing Wu, Xiaohu Guo, Shihai Xing

**Affiliations:** a College of Pharmacy, Anhui University of Chinese Medicine, Hefei, China; b Department of Horticultural Science, University of Minnesota, Minneapolis, MN, USA; c Institute of Traditional Chinese Medicine Resources Protection and Development, Anhui Academy of Chinese Medicine, Hefei, China; d Anhui Province Key Laboratory of Research & Development of Chinese Medicine, Hefei, China

**Keywords:** *Buxus megistophylla*, Buxaceae, chloroplast genome, phylogeny

## Abstract

*Buxus megistophylla* Levl. is one of the most common green horticultural plants in the city presently. Here, we assembled and annotated the complete chloroplast (cp) genome of *B. megistophylla*. The whole length of the genome is 157,611 bp and encodes a total of 124 genes, which contains 89 protein-coding genes, 31 transfer RNA (tRNA) genes, and four ribosomal RNA (rRNA) genes. Phylogenetic tree analysis showed that *B. megistophylla* is separated from two other species of the same family, but these three species, clustered in one clade, are relatively closer to each other compared to the species in other families. This cp genome sequencing and phylogenetic analysis offer genetic background for conservation and may contribute to further evolutionary studies of this species.

*Buxus megistophylla* Levl. is a shrub or small arbor of Buxus family (Jing et al. 2020). It is a temperate and subtropical tree species, widely cultivated in the center and north of China (Zhang et al. 2015). Due to its beautiful tree shape and strong ornamental value, *B. megistophylla* is widely used as an excellent landscaping tree species. It has been used as horticultural plant in the city of south and southwest of China (Hu et al. 2018). Till now, apart from some complete and partial CDS sequences of *B. megistophylla* in public database, there is no information about genome, transcriptome of this plant. We assembled its chloroplast (cp) genome and analyzed its phylogenetic evolution in this study, which would be helpful for better understanding the evolution relationship within the Buxaceae family and further studies on its molecular breeding and genetic engineering.

The fresh young leaves of *B. megistophylla* were collected from Anhui University of Chinese Medicine (117°38′E, 31°93′N), and the specimen was deposited in the Center of Herbarium, Anhui University of Traditional Chinese Medicine, Hefei, China, under accession number 20200819. Genomic DNA was extracted by DNAsecure Plant Kit (TIANGEN Biotech Co., Ltd., Beijing, China), the quality of DNA and its integrity were checked by BioPhotometer Plus (Nucleic acid and protein detector, Eppendorf, Germany). DNA library was constructed from high quality DNA by VAHTSTM Universal DNA Library Prep Kit for IlluminaVR V3 (Vazyme Biotech Co., Ltd., Nanjing, China) and the template size is from 420 bp to 520 bp. Next-generation sequencing was conducted by Suzhou Genewiz Biotechnology Co. Ltd. (Suzhou, China) with an Illumina Hiseq platform (Illumina, San Diego, CA). A total of 3.18 G raw data were obtained after sequencing. The sequencing data quality statistical software Cutadapt (version 1.9.1) (He et al. 2020) was used to filter the contamination, connectors and low-quality sequences, and 3.15 G clean data for subsequent information analysis were obtained. We used the software NOVOPlasty (version 2.7.2) (Dierckxsens et al. 2017) and auxiliary software Spades (Bankevich et al. 2012) to assemble cp genome, and the draft sequences were corrected manually by clean read mapping using bowtie2 (Langmead and Salzberg 2012) and Tablet (Milne et al. 2013). The cp genome of *Buxus microphylla* (GenBank accession no.: NC009599) was used as the reference genome for bioinformatics analysis, and Prodigal (version 3.02) (Hyatt et al. 2010) was used to predict genes.

The complete cp genome of *B. megistophylla* was characterized by using Illumina pair-end sequencing. The cp genome of *B. megistophylla* was 157,611 bp in length, containing a large single-copy region (LSC) of 85,930 bp, a small single-copy region (SSC) of 18,319 bp, and two inverted repeat regions (IRs) of 26,681 bp. The overall GC content is 37.27%, while the corresponding values of the LSC, SSC, and IRs regions are 35.09%, 31.79%, and 42.65%, respectively. GC content of IRs region is the highest. The genome contains totally of 124 genes including 89 coding genes, 31 transfer RNA (tRNA) genes, and four ribosomal RNA (rRNA) genes.

Genome sequences were aligned by using MAFFT (Katoh and Standley 2013), and a maximum-likelihood phylogenetic tree including *B. megistophylla* and other 14 reported species was constructed using MEGA-X (Kumar et al. 2016) with 1000 bootstrap replicates. The result of phylogeny analysis indicated that two species of *Pachysandra terminalis* and *B. microphylla* with cp genome sequenced in family Buxaceae are closely related and clustered under the same node, while *B. megistophylla* is relatively distant from them. But these three, in same family, are relatively closer to each other compared to species of other families, and they are clustered in a clade (Figure 1). From the phylogenetic tree analysis, maybe the traditional classification of species in this family need be modified by molecular biology. This complete cp genome sequence of *B. megistophylla* will provide useful information for future studies in solving the phylogenetic relationships among the species of Buxus family.

**Figure 1.**
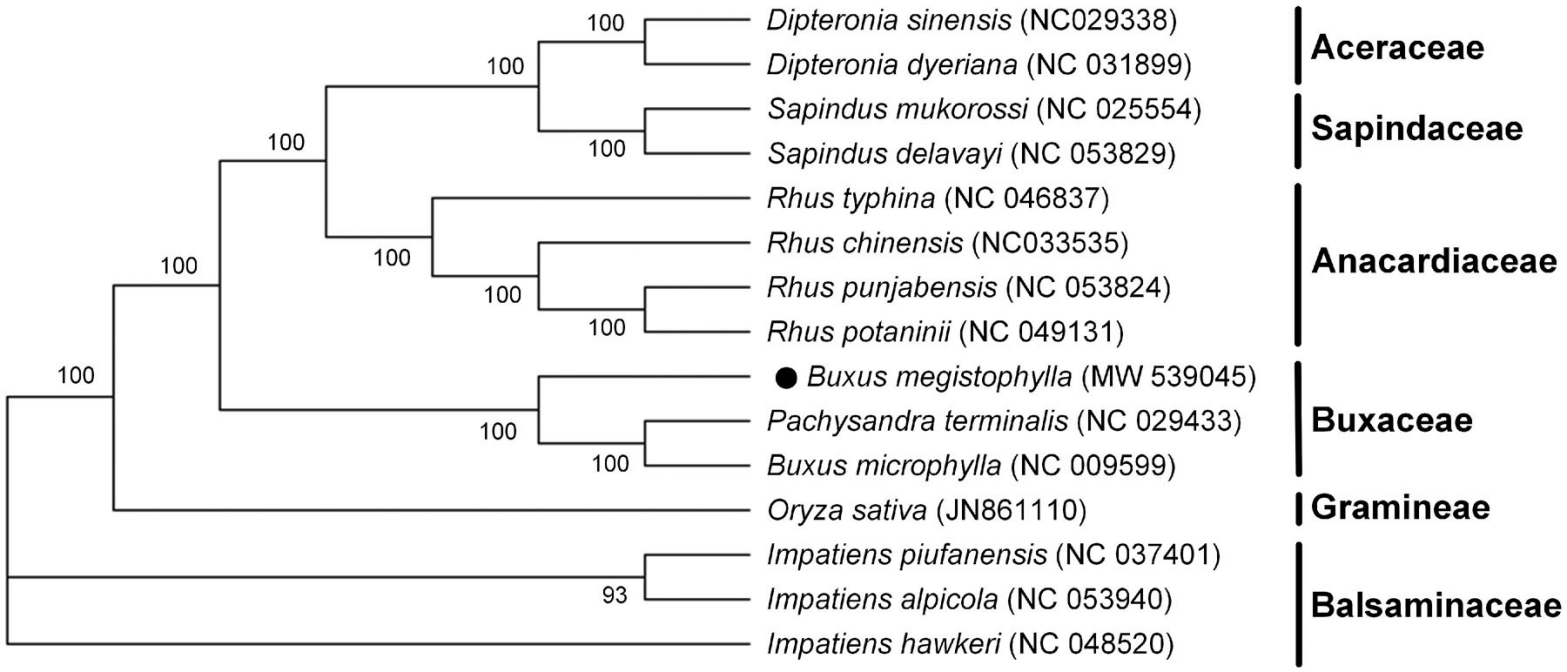
Maximum-likelihood phylogenetic tree resulted from the chosen genome information of 14 relevant chloroplast sequences. Values next to the branches stand for the percentage of replicate trees where the groups clustered together, and the bootstrap parameter was set at 1000 replicates.

## Data Availability

The genome sequence generated during this study is openly available in GenBank of NCBI (https://www.ncbi.nlm.nih.gov/MW539045) under the access number MW539045.1. The other data such as the associated Bio-Project, Bio-Sample, and SRA are at https://www.ncbi.nlm.nih.gov/bioproject/PRJNA734108, https://www.ncbi.nlm.nih.gov/biosample/SAMN19471631, and https://www.ncbi.nlm.nih.gov/sra/SRR14695950, respectively.

## References

[CIT0001] Bankevich A, Nurk S, Antipov D, Gurevich AA, Dvorkin M, Kulikov AS, Lesin VM, Nikolenko SL, Pham S, Prjibelski AD, et al. 2012. SPAdes: a new genome assembly algorithm and its applications to single-cell sequencing. J Comput Biol. 19(5):455–477. 22506599 10.1089/cmb.2012.0021 PMC3342519

[CIT0002] Dierckxsens N, Mardulyn P, Smits G. 2017. NOVOPlasty: de novo assembly of organelle genomes from whole genome data. Nucleic Acids Res. 45(4):e18. 28204566 10.1093/nar/gkw955 PMC5389512

[CIT0003] He B, Zhu R, Yang H, Lu Q, Wang W, Song L, Sun X, Zhang G, Li S, Yang J, et al. 2020. Assessing the impact of data preprocessing on analyzing next generation sequencing data. Front Bioeng Biotechnol. 30(8):817. 10.3389/fbioe.2020.00817 PMC7409520 32850708

[CIT0004] Hu YH, Dou XL, Li JY, Li F. 2018. Impervious surfaces alter soil bacterial communities in urban areas: a case study in Beijing, China. Front Microbiol. 27(9):226. 10.3389/fmicb.2018.00226 PMC5839015 29545776

[CIT0005] Hyatt D, Chen GL, Locascio PF, Land ML, Larimer FW, Hauser LJ. 2010. Prodigal: prokaryotic gene recognition and translation initiation site identification. BMC Bioinformatics. 11:119. 20211023 10.1186/1471-2105-11-119 PMC2848648

[CIT0006] Jing XX, Lun XX, Fan C, Ma WF. 2020. Emission patterns of biogenic volatile organic compounds from dominant forest species in Beijing, China. J Environ Sci. 95:73–81. 10.1016/j.jes.2020.03.049 32653195

[CIT0007] Katoh K, Standley DM. 2013. MAFFT multiple sequence alignment software version 7: improvements in performance and usability. Mol Biol Evol. 30(4):772–780. 23329690 10.1093/molbev/mst010 PMC3603318

[CIT0008] Kumar S, Stecher G, Tamura K. 2016. MEGA7: molecular evolutionary genetics analysis version 7.0 for bigger datasets. Mol Biol Evol. 33(7):1870–1874. 27004904 10.1093/molbev/msw054 PMC8210823

[CIT0009] Langmead B, Salzberg SL. 2012. Fast gapped-read alignment with Bowtie 2. Nat Methods. 9(4):357–359. 22388286 10.1038/nmeth.1923 PMC3322381

[CIT0010] Milne I, Stephen G, Bayer M, Cock PJA, Pritchard L, Cardle L, Shaw PD, Marshall D. 2013. Using Tablet for visual exploration of second-generation sequencing data. Brief Bioinform. 14(2):193–202. 22445902 10.1093/bib/bbs012

[CIT0011] Zhang J, Qin XY, Zhang SD, Xu XS, Pei JP, Fu JJ. 2015. Chemical constituents of plants from the genus *Buxus*. Chem Biodivers. 12(9):1289–1306. 26363874 10.1002/cbdv.201400185

